# Atrasentan increased the expression of klotho by mediating miR-199b-5p and prevented renal tubular injury in diabetic nephropathy

**DOI:** 10.1038/srep19979

**Published:** 2016-01-27

**Authors:** Wen-Ling Kang, Gao-Si Xu

**Affiliations:** 1 Medical Center of the Graduate School, Nanchang University, Nanchang 330000, China; 2 Department of Nephrology, People’s Hospital of Xinyu City, Xinyu 338000, China; 3 Department of Nephrology, Second Affiliated Hospital, Nanchang University, Nanchang 330006 China

## Abstract

Atrasentan is a promising therapy for treating diabetic nephropathy (DN). Here we evaluated whether atrasentan down-regulated the miR-199b-5p expression, thereby increasing klotho and preventing renal tubular injury in DN. One-hundred patients with type 2 diabetes mellitus (T2DM) and 40 healthy subjects were included. A DN mice model was established by an injection of streptozotocin (STZ). Human renal proximal tubular epithelial HK-2 cells were exposed to high glucose (20 mmol/L). Treated the mice and HK-2 cells with atrasentan, and we then investigated whether and how miR-199b-5p and Klotho were involved in preventing renal tubular injury in DN. In patients, the serum miR-199b-5p level increased and the klotho concentration decreased in accordance with elevated albuminuria. Atrasentan down-regulated miR-199b-5p and up-regulated klotho of the DN mice and HK-2 cells exposed to high glucose. High glucose promoted the binding of histone H3 to the miR-199b-5p promoter, and atrasentan canceled this effect. MiR-199b-5p targeted the 3′ UTR of klotho. Overexpression of miR-199b-5p canceled the effects of atrasentan on klotho expression and apoptosis of renal tubular cells in both *in vivo* and *in vitro*. The increased serum klotho, mediated by miR-199b-5p, is a possible mechanism by which atrasentan prevents renal tubular injury in DN.

Diabetic nephropathy (DN) is one of the major microvascular complications of diabetes mellitus (DM) and ultimately results in progressive renal failure^1^. Approximately 20% of patients with DM for 10–20 years will develop DN. Common damages in DN are glomerular injury and tubular injury^2^,^3^. Many studies have examined the tubular damage that occurs in the advanced stages of DN^4^. The renal proximal tubules are uniquely susceptible to a variety of metabolic and hemodynamic factors, which is critical in events leading to apoptosis^4^. Patients with DM show increased proximal and distal tubular epithelia apoptosis due to the proximal tubular epithelial cells under high-glucose ambience^5^. The molecular pathways involved in proximal tubule apoptosis in DN are linked to oxidative stress, inflammation, and fibrosis^6^,^7^. Atrasentan, a selective endothelin-A receptor antagonist, is a promising therapy for reducing residual albuminuria in patients with type 2 DM (T2DM)^8^. A clinical trial of atrasentan is ongoing^9^. However, little is known about the mechanism by which atrasentan protects against tubular injury during the pathological process of DN.

Klotho, originally identified as a product of an antiaging gene in 1997^10^, has been in the spotlight in aging research for more than a decade^11^. Studies have also demonstrated that this antiaging transmembrane protein was also confirmed to be highly expressed in the kidney and presented in the proximal tubule lumen^10^,^12^. In recent years, the roles of klotho in DM and renal diseases have attracted increased attention^13^. Klotho also has been shown to function as an endocrine substance and to have multiple roles in extending lifespan, antioxidation, insulin sensitivity regulation, and stem cell preservation^14^. It was reported that plasma soluble klotho was lower in DM patients than in nondiabetic controls^15^. Extensive studies have also indicated that klotho deficiency in renal tissue and the circulation system is an early event in various models of kidney injury^16^,^17^. Recently, a clinical trial suggested that plasma klotho was inversely correlated with pro-endothelin-1 in T2DM patients.

However, the mechanisms that modulate klotho expression in renal diseases remain to be elucidated. Recently, miR-199b-5p has been found to target klotho in cancer^18^. MicroRNAs (miRNAs) are small noncoding RNAs, which mainly repress their target mRNAs by complementary base pairing and other pathways^19^,^20^. A previous study demonstrated that the genes encoding for miRNAs undergo the same regulatory epigenetic processes as protein coding genes, including DNA methyltransferases, histone deacetylases, and polycomb genes^21^. Liu *et al.* demonstrated that the down-regulation of miR-199a-5p in ovarian cancer was due to promoter hypermethylation. In the present study, we investigated whether the promoter histone deacetylases of miR-199b-5p contributed to the alteration of its expression and whether they were involved in the regulation of klotho in renal tubular injury during DN process. We speculated that regulation of miR-199b-5b and klotho might explain the beneficial effects of atrasentan in the treatment of DN.

## Materials and Methods

### Subjects

Forty healthy volunteers and 100 patients with T2DM were recruited from the Second Affiliated Hospital of Nanchang University. Written informed consent was obtained from all participants. This study was approved by the ethics committee of Nanchang University and carried out in accordance with the guideline of the ethical management. The diagnosis of T2DM was made according to the criteria of the American Diabetes Association^22^. All the T2DM patients were grouped into three groups according to their level of albuminuria: normoalbuminuric subjects (*n* = 35) with an albumin/creatinine ratio lower than 30 μg/mg, microalbuminuric subjects (*n* = 35) with an albumin/creatinine ratio within 30–300 μg/mg in males and within 30–400 μg/mg in females, and macroalbuminuric subjects (*n* = 30) with an albumin/creatinine ratio higher than 300 μg/mg in males and 400 μg/mg in females. The clinical characteristics of all the participants are shown in Table 1.

### Detection of serum klotho

The serum klotho concentration was detected by an ELISA kit (CUSABIO Life science, China) according to manufacturer’s instructions.

### Quantitative PCR

Total RNA was extracted from serum or kidney tissue using Trizol reagent (Invitrogen, USA). Then, 1 μg of total RNA was reverse transcribed to cDNA using a PrimeScript™ Reverse Transcription Kit (TakaRa, Japan) according to the manufacturer’s instructions. Fluorescence quantitative PCR of cDNA was performed with primers specific for miR-199b-5p and klotho expression. All the primers, including stem-loop reverse transcriptase PCR for mature miR-199b-5p, were designed and produced by Shanghai Generay Biotech Co., Ltd. (Shanghai, China). The expression levels of miR-199b-5p and klotho were assessed with a real-time PCR system (Applied Biosystems 7900 Fast Real-Time PCR System, USA) with Power SYBR® Green PCR Master Mix (Applied Biosystems, USA). The real-time PCR procedures were as follows: activation of enzymes at 94 °C for 5 min, 40 cycles of denaturation at 94 °C for 20 s, annealing at 60 °C for 30 s, and extension at 72 °C for 20 s. The quality of the amplification was evaluated by a melting curve. U6 and β-actin served as control genes to normalize the expression of miR-199b-5p and klotho, respectively. The relative expression levels of miR-199b-5p and klotho were calculated with the 2^−ΔΔCT^ method.

### Renal function and TUNEL staining

At week 14, the mice were housed in metabolic cages for collecting urine. The blood was collected and centrifuged to obtain serum at the time of removing the kidneys. Renal function parameters (urinary albumin/creatinine) were detected by high-performance liquid chromatography (HPLC), and serum blood urea nitrogen (BUN), and serum creatinine were detected by ELISA kits (Shanghai Biological Technology Co., Ltd., China). The urine was also collected to determine the indicators of proximal tubular damage such as KIM-1(kidney injury molecule-1), NGAL (neutrophil gelatinase-associated lipocalin) and NAG (N-acetyl-β-D glucosminidase) using ELISA kits (Shanghai Xibao Biological Technology Co., Ltd., China).

TUNEL staining to evaluate the cell apoptosis was performed with an *in situ* cell death detection kit (Roche Applied Science, USA) following the manufacturer’s instructions and as described in a previous study^23^.

### Detection of antioxidant indexes

The antioxidant abilities of kidney mitochondria and HK-2 cells were evaluated with antioxidant indexes, including total antioxidant capacity (T-AOC), superoxide dismutase (SOD), catalase (CAT), and reduced glutathione (GSH). The concentrations of T-AOC, SOD, CAT, and GSH were determined with specific commercial kits (Nanjing Jiancheng Bioengineering Institute, China) according to the manufacturer’s instructions.

### Western blot

The kidney tissues were homogenized on ice in phosphate-buffered saline (PBS) to obtain the homogenate. The total protein from kidney homogenate and HK-2 cells were isolated with an RIPA lysis buffer (Beyotime, China). The supernatants were collected after centrifugation at 10,000 × g at 4 °C for 15 min. The protein concentration was detected with the BCA method. Thirty micrograms of protein from each sample were run on 10% SDS-PAGE electrophoresis and then transferred to PVDF membranes and blocked in 5% nonfat milk prior to incubation with the primary anti-klotho antibody (Cell Signaling Technology, USA) at a dilution of 1:1000 overnight at 4 °C. After being washed, the membrane was incubated with horseradish peroxidase-conjugated secondary antibody for 1 h at room temperature. The antigen-specific signal was then detected by incubation with enhanced chemoluminescence substrate (Pierce Biotechnology). The bands were visualized using a BeyoECL Plus ECL Kit (Beyotime, China). GAPDH severed as a control protein to normalize klotho expression.

### Mitochondrial transmembrane potential and mitochondria morphology

The mice were administered 0.35 mL/100 g of chloral hydrate to induce narcosis. The abdominal cavity was exposed, and the fresh kidney was isolated. After washing with ice and normal saline and removing the renal capsule, the renal hilum connective tissue and blood vessels and kidney mitochondria were isolated by differential and sucrose density centrifugation, as reported previously^24^. The mitochondrial transmembrane potential was assessed using a Mitochondrial Membrane Potential assay Kit with JC-1 (Beyotime, China). JC-1, a fluorochrome, can be accumulated in the mitochondrial matrix to form polymer, which gives off a strong red fluorescence (Ex = 585 nm, Em = 590 nm). JC-1 exists in the form of monomer in the cytoplasm of unhealthy cell, and gives of a green fluorescence (Ex = 514 nm, Em = 529 nm).

Transmission electronic microscopy analysis was performed to observe the morphology of kidney mitochondria. Fresh kidney tissue was rapidly removed after sacrificing the mice and washed with a phosphate buffer (PBS, pH 7.4). Renal tubular epithelial cells were removed^25^ and fixed with 2.5% glutaraldehyde for 2 h and then rinsed with PBS. Osmium tetroxide (1%) was used to fix the cells for another 2 h. After washing with PBS, the samples were dehydrated and embedded in Durcupan resin (Sigma-Aldrich, USA). Ultrathin sections were cut and observed with a HT7700 transmission electron microscope (Hitachi, Japan).

### Cell culture

Human renal proximal tubular epithelial HK-2 cells were purchased from the American Type Culture Collection. The HK-2 cells were cultured in DMEM (Sigma-Aldrich, USA), supplemented with 10% fetal bovine serum, 100 U/mL of penicillin, and 100 μg/mL of streptomycin (Sigma-Aldrich, USA) in a humidified atmosphere containing 5% CO_2_ at 37 °C. The medium for the control cells contained 5 mmol/L of glucose. In the experimental group, the medium was supplemented with 20 mmol/L of glucose for 72 h to produce a high-glucose model. The cells in logarithmic growth phase were used in subsequent experiments.

### Establishment of a diabetic mouse model

One-hundred C57BL/6 mice (female/male = 1/1) aged 6–8 weeks were purchased from Shanghai Laboratory Animal Center of the Chinese Academy of Sciences (Shanghai, China). All the animal experimental protocols were approved by the Animal Care and Use Committee of the Second Affiliated Hospital, Nanchang University. A DN mouse model was induced by a single intraperitoneal injection of streptozotocin (STZ, 120 mg/kg) for 2 days. Control mice received an equal volume of a sodium citrate vehicle buffer. Eight weeks after the injection, all the mice were positive for albuminuria. The diabetic mice were randomly divided and received either 5 mg/kg of atrasentan or saline by gavage administration per day for 8 weeks. The mice treated with atrasentan were also injected intravenously with AAV- miR-199b-5p (with or without 20 mg/kg klotho by intraperitoneal injection) or a control vector twice per week for 8 weeks. In addition,

### Luciferase reporter assay

Fragments of the 3′ UTR of klotho containing putative miR-199b-5p binding sites were amplified by PCR technology and then cloned into pMIR- RB-REPORTTM vectors (Guangzhou RiboBio Co., Ltd., China). The cells were grown to 50% confluence in 12-well plates. The constructs were then co-transfected with pre-mir-199b-5p or a negative control (NC) with Lipofectamine2000 (Invitrogen, USA) according to the manufacturer’s recommendations. After 48 h of transfection, luciferase activity was measured with a Dual-Luciferase Reporter Assay System Kit (Promega, USA).

### Chromatin immunoprecipitation (ChIP) assay

A chromatin immunoprecipitation (ChIP) Assay Kit (Upstate Biotechnology, USA) was used to assess DNA-protein interactions at the miR-199b-5p promoter sequences. Briefly, the cells were harvested and fixed with 1% (v/v) formaldehyde at room temperature for 10 min to cross-link the protein-DNA complexes. After washing with ice-cold PBS containing protease inhibitors, the cells were resuspended in a ChIP lysis buffer containing 1% SDS for 10 min on ice, and lysates were sonicated to shear DNA fragments to lengths from 200 to 1000 bp. The chromatin was then immunoprecipitated with an equal amount of antibodies against histone H3, histone H4, or control IgG overnight at 4 °C. Protein-DNA-antibody complexes were precipitated with protein A-agarose beads for 2 h at 4 °C. Input or DNA in the complex was subjected to quantitative PCR.

### Statistical analysis

The statistical analyses were performed using SPSS 15.0 software. A one-way ANOVA with Turkey’s multiple comparison tests was used to analyze data with more than two groups. Two-way analysis of variance followed by Bonferroni’s post hoc test was performed to compare the values of urinary albumin/creatinine concentrations among three groups during the treatment of atrasentan. Differences between two groups were analyzed by an independent Student’s t test. The data were expressed as the mean ± SD. Significance was considered at values of *P* < 0.05.

## Results

### Characteristics of the participants

The general characteristics of the participants are presented in Table 1. Albumin, BUN, and creatinine are commonly used to evaluate renal function. Here, we grouped the diabetes patients according to their level of albuminuria. The results demonstrated that there were no differences among the four groups in age, gender, BMI, and blood lipid concentrations. The macroalbuminuric subjects had significantly higher serum BUN and creatinine levels than the normal, normoalbuminuric, or microalbuminuric ones. Serum miR-199b-5p levels were significantly decreased in the subjects with abnormal albuminuria and the highest level was in the macroalbuminuric subjects. The circulating concentration of klotho decreased with an increased level of albuminuria. Univariate correlations with serum miR-199b-5p level were also performed. As shown in Table 2, the serum miR-199b-5p level was negatively related to serum BUN, creatinine, and klotho concentrations.

### Atrasentan altered the miR-199b-5p and klotho expression levels of STZ-induced DN mice

**Figure 1:**
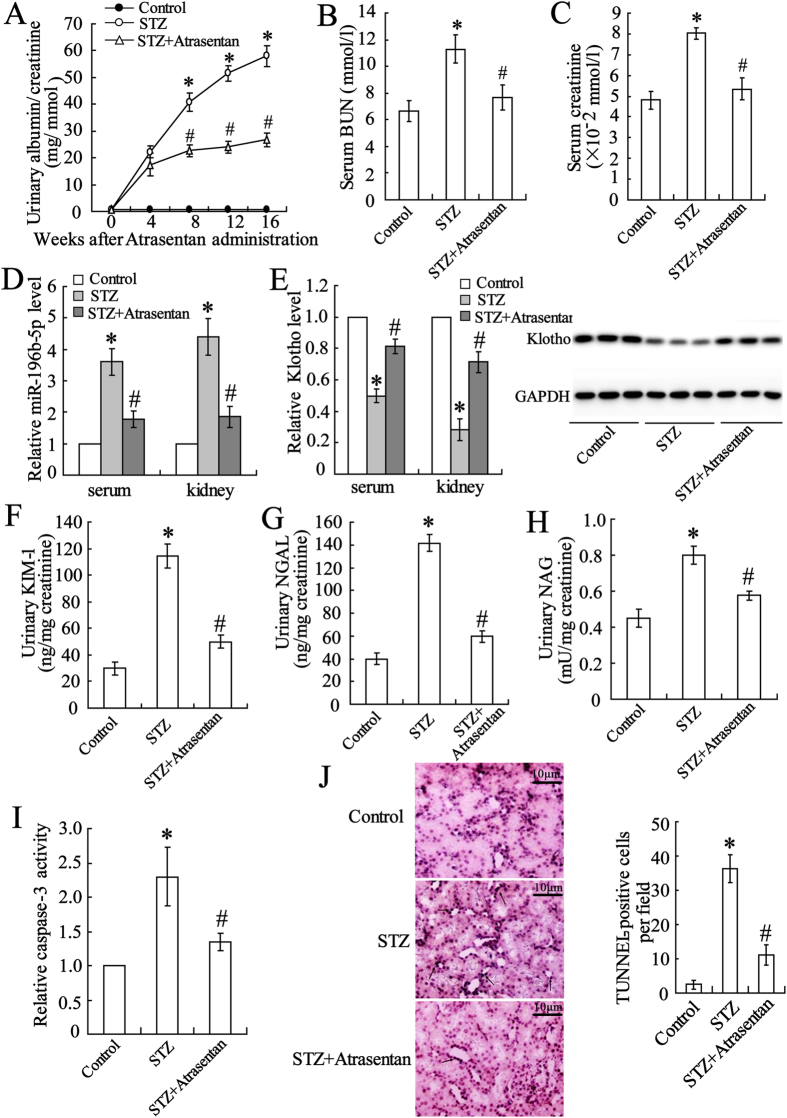
Atrasentan improved renal function, decreased miR-199b-5p expression, and increased klotho expression in STZ-induced DN mice. The urinary albumin/creatinine (**A**), serum BUN (blood urea nitrogen) (**B**), and serum creatinine (**C**) were measured to evaluate renal function. Real-time PCR was used to detect serum and renal tubular epithelial cell miR-199-5p expression (**D**). The serum klotho level was measured with an ELISA kit, and the renal klotho level was detected with real-time PCR and a Western blot (**E**). The levels of urinary KIM-1(kidney injury molecule-1) (**F**), NGAL (neutrophil gelatinase-associated lipocalin) (**G**) and NAG (N-acetyl-β-D glucosminidase) (**H**) were measured by ELISA kits. A Caspase-3 activity assay (**F**) and a TUNEL assay (**G**) were used to assess the apoptosis of renal tubular epithelial cells of mice. *n* = 10. *P < 0.05 versus the control group; ^#^*P* < 0.05 versus the STZ-induced DN group.

The DN mouse model was established by administration of STZ. There was no difference between the control and DN mice in body weight, but the blood glucose was significantly elevated in the DN mice (Suppl. 1). The renal function was then evaluated. As shown in Fig. 1A–C, the concentrations of urinary albumin/creatinine, serum BUN, and serum creatinine were significantly elevated in the DN mice, and atrasentan markedly decreased these values. The levels of miR-196-5p and klotho in the serum and renal tubular epithelial cells of the DN mice were detected with real-time PCR and a Western blot. The results showed that the expression of miR-196-5p was up-regulated, whereas that of klotho was down-regulated. Atrasentan reversed the alterations in the expression of miR-196-5p and klotho (Fig. 1D,E). The indicators of proximal tubular damage urinary KIM-1, NGAL and NAG were also evaluated. The data showed that the concentrations of these data were also prominently increased in STZ induced mice, while atrasentan dramatically decreased these values (Fig. 1F–H). Caspase-3 activity and TUNEL assays were used to assess renal tubular epithelial cell apoptosis. As shown in Fig. 1I,J cell apoptosis was profoundly elevated and largely inhibited by atrasentan.

### Atrasentan improved the renal mitochondrial function of STZ-induced diabetic mice

**Figure 2:**
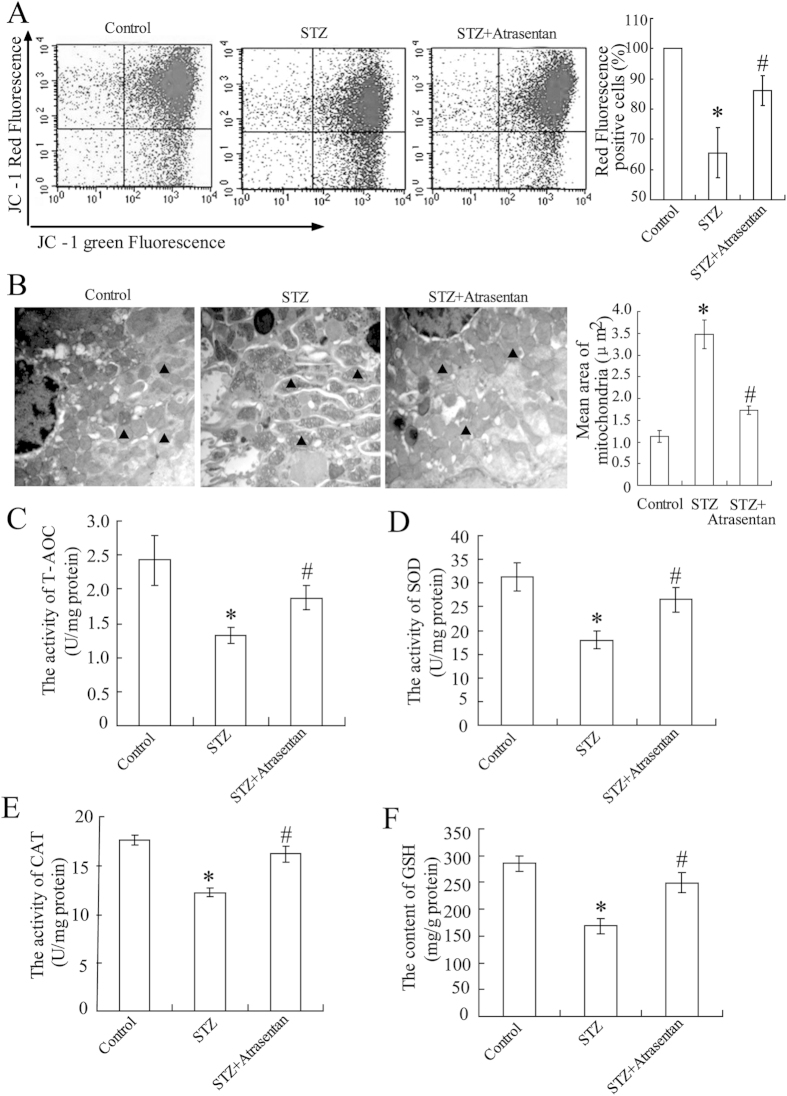
Atrasentan elevated renal mitochondria membrane potential and increased the mitochondrial antioxidant ability of STZ-induced diabetic mice. The renal mitochondria membrane potential was detected with a commercial kit (**A**). Transmission electronic microscopy analysis was used to observe the kidney mitochondria morphology, and mitochondria was labelled with a black solid triangle (**B**). Levels of antioxidant indicators (T-AOC, SOD, CAT, and GSH) of renal mitochondria were detected by commercial kits in STZ-induced mice (**C–F**). *n* = 10 *P < 0.05 versus the control group; ^#^*P* < 0.05 versus the STZ-induced diabetic group.

In the STZ-induced diabetic mice, a significant decrease in renal mitochondria membrane potential (Fig. 2A) and mitochondrial swelling (Fig. 2B) was observed. The values of antioxidant indicators (T-AOC, SOD, CAT, and GSH levels) in renal mitochondria also decreased (Fig. 2C–F). Atrasentan reversed these alterations, elevating the renal mitochondrial membrane potential, inhibiting mitochondrial swelling, and enhancing the antioxidant ability of the mitochondria.

### Atrasentan altered the expression of miR-199b-5p and klotho and the antioxidant ability of HK-2 cells

**Figure 3:**
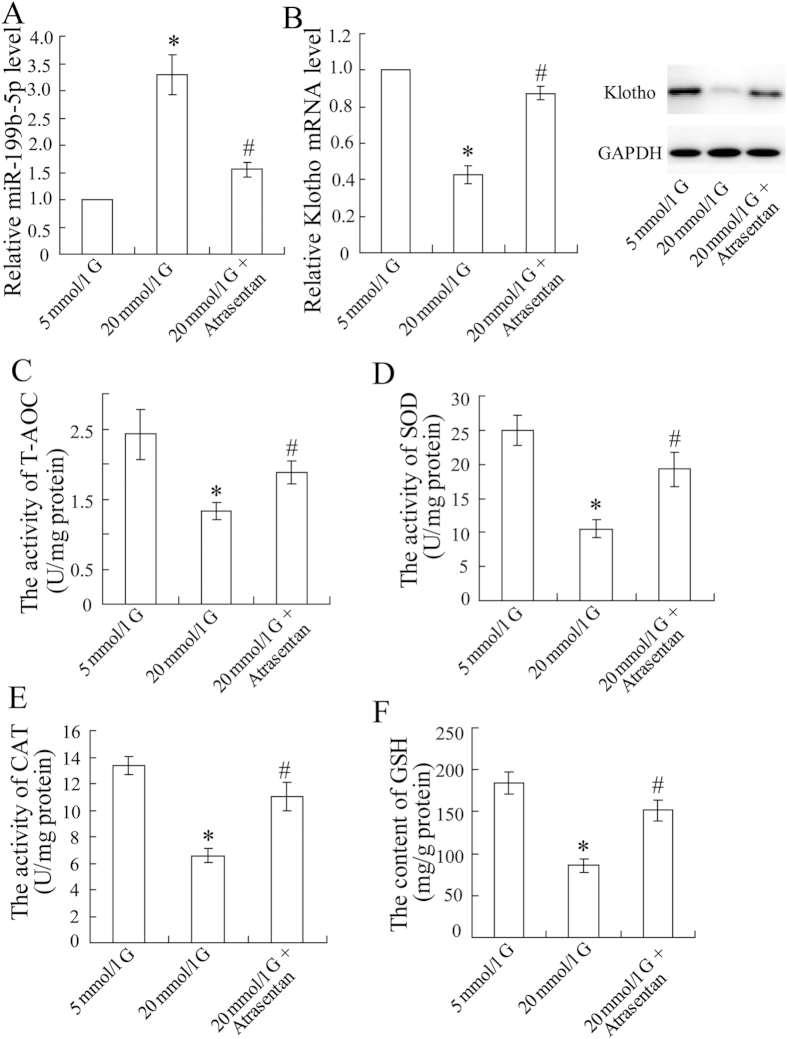
Atrasentan up-regulated miR-199b-5p expression, down-regulated klotho expression, and increased the antioxidant ability of HK-2 cells exposed to a high concentration of glucose. The HK-2 cells were incubated with different concentrations of glucose (5 mmol/L or 20 mmol/L) for 72 h and treated with atrasentan. The expression of miR-199b-5p was quantified by real-time PCR (**A**). The expression of klotho was measured by real-time PCR and a Western blot (**B**). Levels of antioxidant indicators (T-AOC, SOD, CAT, and GSH) of renal mitochondria in HK-2 cells were detected by commercial kits (**C–F**). *P < 0.05 versus the cells exposed to 5 mmol/L glucose; ^#^*P* < 0.05 versus the cells exposed to 20 mmol/L glucose.

To investigate the role and mechanism of miR-199b-5p and klotho in DN, *in vitro* experiments were performed. Increased miR-199b-5p expression (Fig. 3A) and decreased klotho expression (Fig. 3B) were observed in the HK-2 cells exposed to a high concentration (20 mmol/L) of glucose. The concentrations of the antioxidant indicators T-AOC, SOD, CAT, and GSH also decreased in the cells exposed to high glucose (Fig. 3C–F). As expected, these changes were reversed by the supplementation with atrasentan.

### Atrasentan altered the epigenetic modification of the miR-199b-5p promoter in HK-2 cells

**Figure 4:**
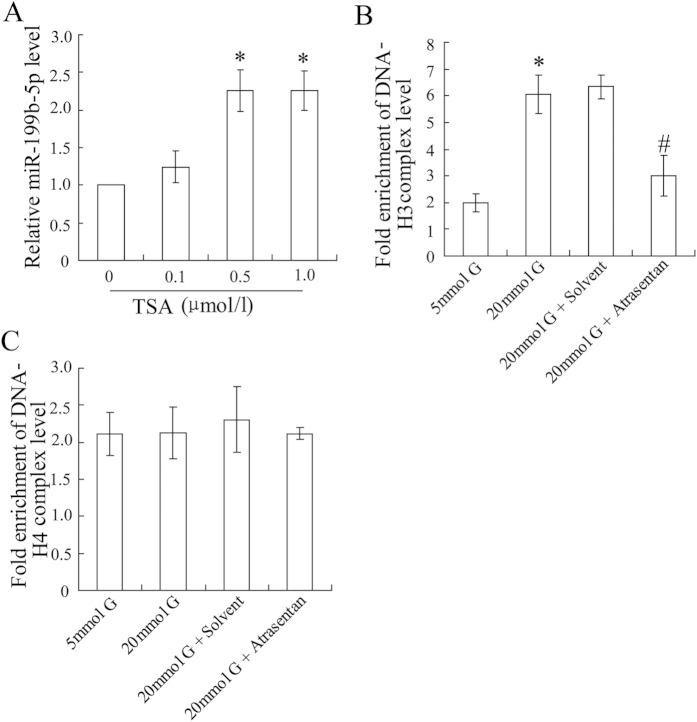
Atrasentan decreased histone deacetylation of the miR-199b-5p promoter in HK-2 cells exposed to high glucose. The HK-2 cells were exposed to various concentrations of TSA (0.1, 0.5 and 1.0 μmol/L) for 72 h and the expression of miR-199b-5p was then quantified by real-time PCR (**A**). The HK-2 cells were incubated with various concentrations of glucose (5 mmol/L or 20 mmol/L) and treated with atrasentan. DNA-H3 and DNA-H4 complex levels were detected by a ChIP assay (**C**,**D**). *P < 0.05 versus the cells exposed to 0 μmol/L TSA or 5 mmol/L glucose; ^#^*P* < 0.05 versus the cells exposed to 20 mmol/L glucose and solvent.

The epigenetic modification of the miR-199b-5p promoter was explored in HK-2 cells. Administrated cells with methylase 5′-Aza-dC, no alteration of miR-199b-5p expression was observed (data not shown). However, when cells were co-incubated with the histone deacetylase inhibitor Trichostatin A (TSA), miR-199b-5p expression levels were up-regulated at higher levels (Fig. 4A). The ChIP assay demonstrated that a high concentration of glucose elevated the binding of histone H3 to the miR-199b-5p promoter and that atrasentan canceled this effect (Fig. 4B). In addition, the results indicated that histone H4 was not involved in this process (Fig. 4C).

### 3.6 miR-199b-5p targeted klotho and regulated its expression in HK-2 cells

**Figure 5:**
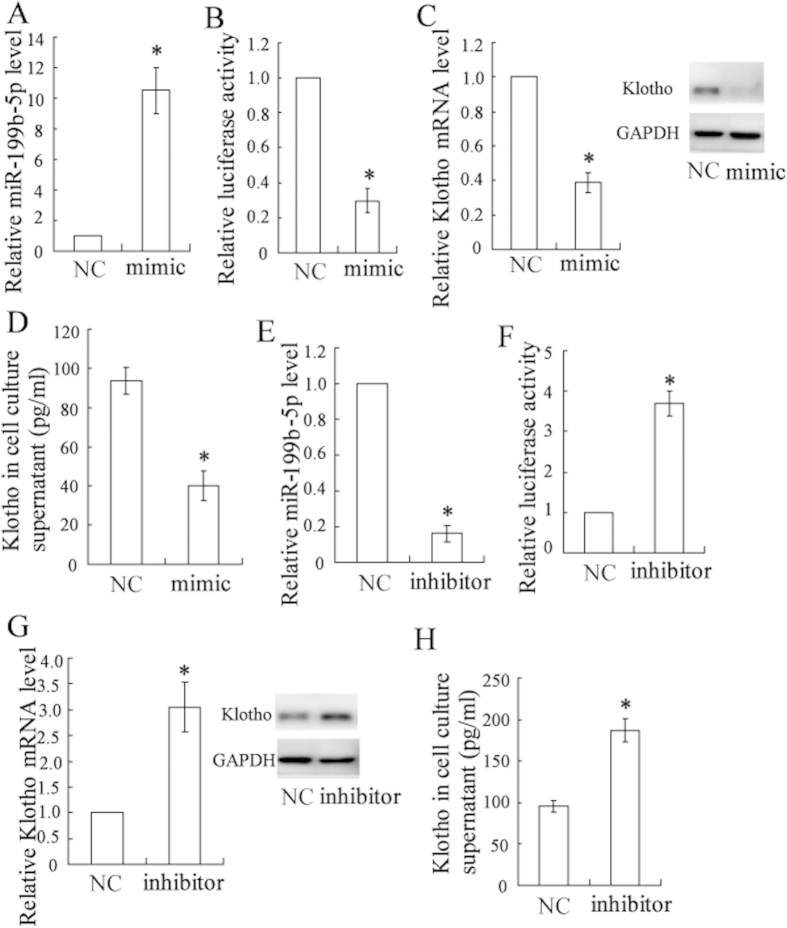
miR-199b-5p targeted klotho and regulated its expression in HK-2 cells. The HK-2 cells were transfected with an miR-199b-5p mimic/inhibitor or NC, and the expression of miR-199b-5p was quantified by real-time PCR (**A,E**). The 3′ UTR activity of klotho was detected by a luciferase reporter assay (**B,F**). The expression of klotho was quantified by real-time PCR and a Western blot (**C,G**). The klotho concentration of cell culture supernatant was determined with an ELISA kit (**D**,**H**). *P < 0.05 versus the cells transfected with the NC.

**Figure 6:**
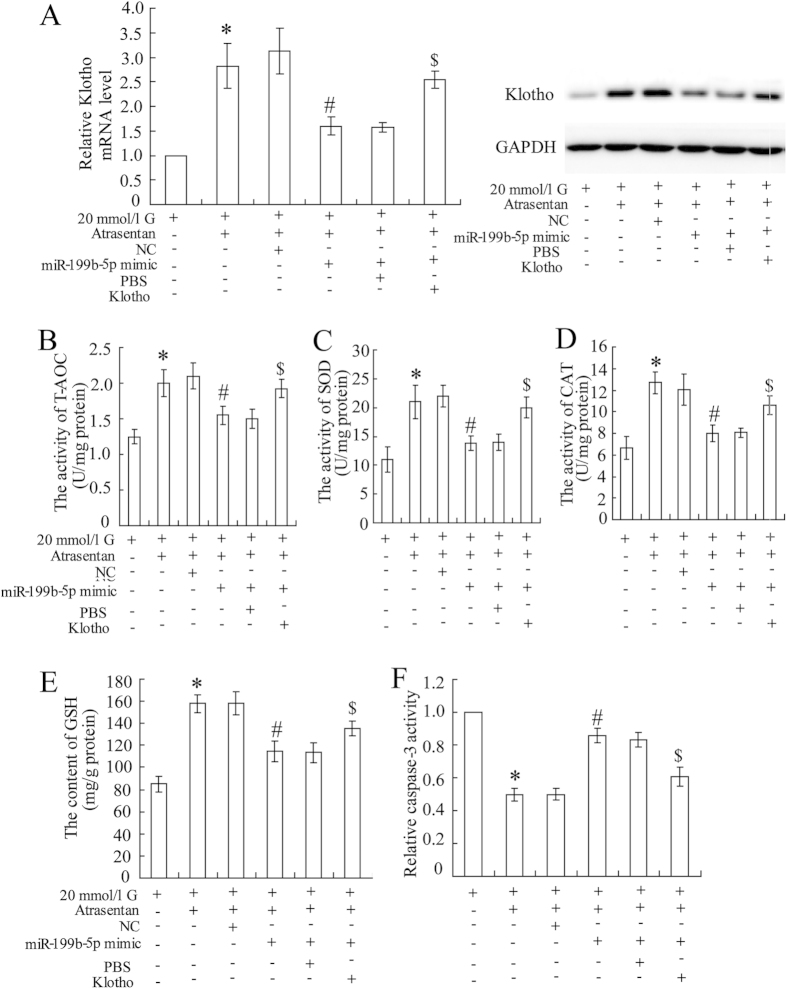
miR-199b-5p participated in the effects of atrasentan on the expression of klotho, antioxidant activities, and caspase activity of HK-2 cells. The HK-2 cells were treated with 20 mmol/L glucose and co-incubated with atrasentan. An miR-199b-5p mimic (100 mmol) or an NC was then transfected into the cells. The concentration of klotho treated in cells was 20 nmol/L. The expression of klotho was measured by real-time PCR and a Western blot (**A**). Levels of antioxidant indicators (T-AOC, SOD, CAT, and GSH) of renal mitochondria were detected by commercial kits in the HK-2 cells (**B–E**). The activity of caspase-3 was detected with an ELISA kit (**F**). *P < 0.05 versus cells treated with 20 mmol/L glucose; ^#^*P* < 0.05 versus the cells treated with 20 mmol/L glucose + atrasentan + NC; ^$^*P* < 0.05 versus the cells treated with20 mmol/L glucose + atrasentan + miR-199b-5p mimic + PBS.

As reported in the MicroRNA.org database, klotho is a potential target of miR-199b-5p (Suppl. 1). To further demonstrate the relationship between miR-199b-5p and klotho, the HK-2 cells were transfected with a miR-199b-5p mimic or inhibitor to overexpress or down-regulate the expression of miR-199b-5p (Fig. 5A,E). A luciferase reporter assay showed that the miR-199b-5p mimic significantly decreased the 3′ UTR activity of klotho (Fig. 5B) and inhibited its expression (Fig. 5C,D), whereas the miR-199b-5p inhibitor had the opposite effects (Fig. 5F–H). The miR-199b-5p mimic reversed the atrasentan-induced increase in the expression of klotho (Fig. 6A). The miR-199b-5p mimic also canceled the elevated activities of the antioxidant indicators (T-AOC, SOD, and CAT, and GSH) and the reduced caspase-3 activity induced by atrasentan (Fig. 6B–F). Interestingly, the effects of the miR-199b-5p mimic on klotho expression, antioxidant ability, and caspase-3 activity were all dramatically reversed in the HK-2 cells treated with 20 mmol/L glucose.

### The effects of miR-199b-5p and klotho on renal function *in vivo*

**Figure 7:**
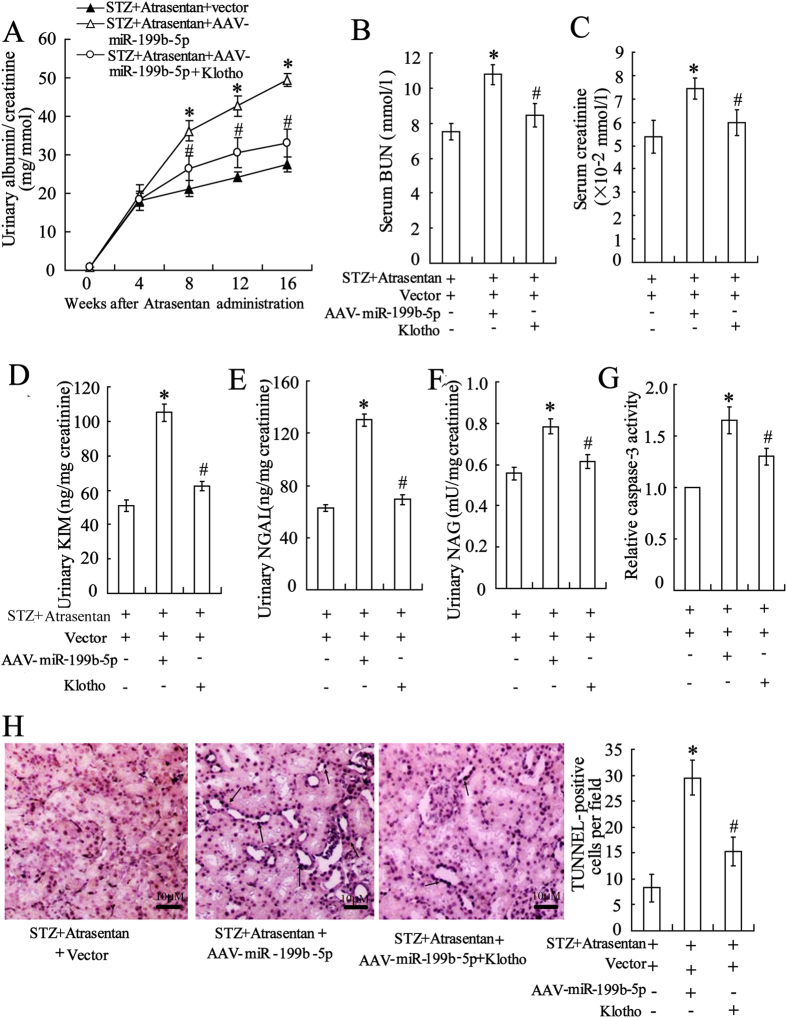
The effects of miR-199b-5p and klotho on the renal function of STZ-induced DN mice. The STZ-induced DN mice were treated with 5 mg/kg/day of atrasentan by intragastric administration for 8 weeks. An AAV-miR-199b-5p/empty vector, with or without 20 mg/kg of klotho by intraperitoneal injection, was intravenously injected into the mice twice every week for 8 weeks. The urinary albumin/creatinine (**A**), serum BUN (blood urea nitrogen) (**B**), and serum creatinine (**C**) were detected to evaluate the renal function of the mice. The levels of urinary KIM-1(kidney injury molecule-1) (**D**), NGAL (neutrophil gelatinase-associated lipocalin) (**E**) and NAG (N-acetyl-β-D glucosminidase) (**F**) were measured by ELISA kits. A caspase-3 activity assay (**G**) and a TUNEL assay (**H**) were used to assess the apoptosis of renal tubular epithelial cells of the mice. *n* = 10. *P < 0.05 versus the STZ + atrasentan + vector group; ^#^*P* < 0.05 versus the STZ + atrasentan + AAV-miR-199b-5p group.

To investigate the effects of miR-199b-5p and klotho on renal function *in vivo*, the STZ-induced diabetic mice were injected with AAV-miR-199b-5p to overexpress miR-199-5b. As shown in Fig. 7, overexpressed miR-199b-5p resulted in an increase in renal function parameters such as urinary albumin/creatinine, serum BUN, serum creatinine, urinary KIM-1, urinary NGAL and urinary NAG (Fig. 7A–F). It also led to an increase in caspase-3 activity and TUNEL-positive cells (Fig. 7G,H) in the DN mice treated with atrasentan. As expected, the additional injection of klotho reversed the negative effects of AAV-miR-199b-5p on renal function *in vivo*.

## Discussion

In current study, we focused on the function of miR-199b-5p in the regulation of klotho expression in renal tubular injury of DN. We speculated that it may be one mechanism by which atrasentan exerts its effects in the treatment of DN. We observed that high glucose increased histone H3 acetylation in the miR-199b-5p promoter region, which led to the activation of this miRNA. MiR-199b-5p also targeted klotho and down-regulated its expression, illustrating a potential mechanism for the protective effect of atrasentan against renal tubular injury in DN.

Many mechanisms have been proposed to explain the pathogenesis of renal injury in DN^26^. One mechanism, which is supported by increasing evidence, is that oxidative stress plays a critical role in the onset and progression of diabetes and DN^27^. It is known that high glucose uptake in diabetes overwhelms the mitochondrial electron transport system and induces overproduction of reactive oxygen species, which result in oxidative stress and cell apoptosis in proximal tubular cells^28^,^29^. High glucose may also serve as an initiating factor and be directly responsible for the causation of tubular damage and apoptosis in DN^4^. In the STZ-induced DN mice, atrasentan elevated the renal mitochondria membrane potential and increased the mitochondrial antioxidant ability. In addition, it markedly decreased indicators levels of glomerular damage and proximal tubular damage and inhibited the apoptosis of renal tubular epithelial cells, indicating that atrasentan is a potential therapy for treating DN.

Klotho exerts pleiotropic biological actions, and it is expressed in multiple tissues and organs. The highest expression of klotho has been reported in the kidney^10^. Recent studies demonstrated that klotho protected organs and cells against damage and apoptosis by increasing the endogenous antioxidative capacity^30^,^31^,^32^. Another study reported that klotho deficiency was potentially linked to enhanced oxidative stress in patients with end-stage renal disease^33^. The increased level of thioredoxin/peroxiredoxin system with the greatest effect on the induction of Prx-2, an antioxidant enzyme has been revealed to be one possible mechanism of the antioxidant effects of Klotho^34^. However, the regulation of Klotho expression remains unclear. In this study, we observed that the circulating klotho level was significantly reduced in abnormal abuminuric patients with T2DM and that klotho levels in plasma and renal tubular epithelial cells were reduced in the STZ-induced DN mice. Lee *et al.* reported that α-klotho levels in plasma and urine were higher in diabetes patients with relatively preserved renal function than in nondiabetic controls^35^. Another study found that circulating klotho levels were significantly decreased in patients with glycated hemoglobin (HbA1c) of ≥6.5% compared with those with HbA1c of <6.5%^36^. Kacso *et al.* demonstrated that the expression of klotho was reduced in patients with early chronic kidney disease but increased in diabetic patients^37^, which may partially interpret the conflicting data. The dynamic alteration of klotho in different pathological conditions is very complex, and more studies are needed to illustrate this process. However, most researchers consider that the production of klotho is decreased in chronic renal failure and that its deficiency is closely related to the development and progression of renal diseases^12^. In the present study, atrasentan elevated serum levels of klotho in the renal tubular epithelial cells of STZ-induced DN mice and increased its expression in the HK-2 cells exposed to high glucose.

Klotho has been shown to have a major pathophysiological role in kidney diseases^13^. However, little is known about the regulatory mechanism of klotho in DN. Recently, He *et al.* found that klotho was a target gene of miR-199a-5p in cancer^18^, suggesting that the miR-199 family may be involved in the regulation of klotho in DN. We also found that miR-199b-5p targeted klotho at two binding sites using the MicroRNA.org data bank and that the activation of miR-199b-5p inhibited the 3′ UTR activity of klotho and down-regulated its expression level in HK-2 cells. In a clinical trial, serum miR-199b-5p was significantly elevated in T2DM subjects with abnormal albuminuria compared with patients with normal albuminuria, and its level was positively associated with serum BUN and creatinine. A previous study reported that serum miR-199a was up-regulated in diabetic mice and that this resulted in an increase in beta cell apoptosis^38^. Another study of T2DM patients found that plasma miR-199a was overexpressed and that it played a role in repressing glucose uptake^39^. In the present study, high glucose (20 mmol/L) significantly increased the expression level of miR-199b-5p in HK-2 cells. Importantly, we found that high glucose enhanced histone H3, but not H4, binding to the miR-199b-5p promoter and that atrasentan markedly weakened this binding. Combined with the decreased antioxidant abilities levels in the cells exposed to 20 mmol/L glucose, we hypothesized that high glucose leads to excessive production of reactive oxygen species and depletion of antioxidant enzymes, resulting in oxidative stress, up-regulation of histone acetylation, and activation of miR-199a-5p. Atrasentan led to a decrease in histone acetylation, suggesting one possible mechanism by which it may down-regulate miR-199b-5p. In STZ induced diabetics mice, additional injection of klotho reversed the negative effects of AAV-miR-199b-5p on renal function. However, lack of 100% recovery of renal function suggests that atrasentan may have other effects independent of klotho expression that could be important for its preventive function.

The data from the present study suggest that atrasentan may be an effective therapy for treating DN. The increase in the level of klotho, mediated by miR-199b-5p, may be a possible mechanism by which atrasentan prevents renal tubular injury in DN.

## Additional Information

**How to cite this article**: Kang, W.-L. and Xu, G.-S. Atrasentan increased the expression of klotho by mediating miR-199b-5p and prevented renal tubular injury in diabetic nephropathy. *Sci. Rep.* **6**, 19979; doi: 10.1038/srep19979 (2016).

## Supplementary Material

Supplementary Information

## Figures and Tables

**Table 1:** Subjects characteristics.

Parameter	Normal (n = 40)	Normoalbuminuric (n = 35)	Microalbuminuric (n = 35)	Macroalbuminuric (n = 30)
Age (years)	51.5 ± 2.3	50.8 ± 2.1	51.3 ± 2.2	52.5 ± 2.6
Gender, male (%)	67%	65%	69%	71%
BMI (kg/m^2^)	21.9 ± 2.4	23.6 ± 3.5	23.7 ± 3.4	22.7 ± 2.7
TC (mmol/l)	5.2 ± 0.37	5.4 ± 0.41	5.5 ± 0.45	5.5 ± 0.46
HDL-C (mmol/l)	1.35 ± 0.07	1.29 ± 0.08	1.21 ± 0.07	1.23 ± 0.08
LDL-C (mmol/l)	2.53 ± 0.15	2.57 ± 0.17	2.59 ± 0.18	2.61 ± 0.19
TGs (mmol/l)	1.68 ± 0.19	1.79 ± 0.2	1.89 ± 0.22	1.92 ± 0.25
SBP (mmHg)	122.5 ± 4.1	123.7 ± 4.1	128.4 ± 5.1	135.3 ± 5.2*
DBP (mmHg)	85.2 ± 2.3	85.6 ± 3.2	87.2 ± 3.4	90.7 ± 3.5*
FBG (mmol/l)	5.24 ± 0.23	9.23 ± 0.57*	9.91 ± 0.69*	10.51 ± 0.81*
HbA_1c_ (%)	5.35 ± 0.45	8.1 ± 0.69*	8.21 ± 0.75*	8.31 ± 0.79*
BUN (mmol/l)	4.98 ± 1.31	5.14 ± 1.4	6.21 ± 1.7	9.98 ± 3.2*^#$^
Creatinine (mg/dl)	64.1 ± 6.5	65.3 ± 6.8	67.5 ± 7.7	126.7 ± 19.5*^#$^
miR-199b-5p (×10^-3^)	1.1 ± 0.13	1.9 ± 0.21*	2.1 ± 0.27*	3.5 ± 0.3*^#$^
Klotho (pg/ml)	470 ± 45	400 ± 31*	350 ± 30*	321 ± 28*^#$^

Data are presented as mean ± SD. *P < 0.05 versus control, ^#^P < 0.05 versus normoalbuminuric, ^$^P < 0.05 versus microalbuminuric. Abbreviations: BMI, body mass index; TC, total cholesterol; HDL-C, high density lipoprotein cholesterol; LDL-C, low density lipoprotein cholesterol; TGs, triglycerides; SBP, systolic blood pressure; DBP, diastolic blood pressure; FBG, fasting blood glucose; HbA_1c_, glycated hemoglobin; BUN, blood urea nitrogen.

**Table 2:** Univariate correlations with serum miR-199b-5p levels concentrations.

Parameter	r	P
Age	0.09	0.18
Gender	−0.11	0.16
BMI	0.082	0.31
TC	0.091	0.17
HDL-C	−0.13	0.11
LDL-C	0.12	0.12
TGs	0.17	0.09
SBP	0.18	0.08
DBP	0.18	0.08
FBG	0.2	0.072
HbA_1c_	0.25	0.065
BUN	0.42	<0.001
Creatinine	0.45	<0.001
Klotho	−0.54	<0.001

Abbreviations: BMI, body mass index; TC, total cholesterol; HDL-C, high density lipoprotein cholesterol; LDL-C, low density lipoprotein cholesterol; TGs, triglycerides; SBP, systolic blood pressure; DBP, diastolic blood pressure; FBG, fasting blood glucose; HbA1c, glycated hemoglobin; BUN, blood urea nitrogen.
