# Corrigendum: Atrasentan increased the expression of klotho by mediating miR-199b-5p and prevented renal tubular injury in diabetic nephropathy

**DOI:** 10.1038/srep46965

**Published:** 2018-04-06

**Authors:** Wen-Ling Kang, Gao-Si Xu

Scientific Reports 6: Article number: 19979; 10.1038/srep19979 published online: 01 27 2016; updated: 04 06 2018.

The original version of this Article contained errors in the affiliation list where Wen-Ling Kang was inadvertently affiliated to ‘Medical Center of the Graduate School, Nanchang University, Nanchang 330000, China’ instead of the ‘Department of Nephrology, the Second Affiliated Hospital, Nanchang University, Nanchang 330006 China’. These errors have now been corrected in the HTML and PDF versions of your Article, and in the accompanying Supplementary Information.

