# Supplementary material for: Atrasentan increased the expression of klotho by mediating miR-199b-5p and prevented renal tubular injury in diabetic nephropathy
Source: Sci Rep. 2016 Jan 27;6:19979. doi: 10.1038/srep19979 (PMC4728478; doi:10.1038/srep19979)
Supplement: Supplementary Information [file srep19979-s1.pdf]

Atrasentan increased the expression of klotho by mediating miR-199b-5p and prevented renal tubular injury in diabetic nephropathy

Wen-Ling Kang [1,2], Gao-Si Xu [3*]

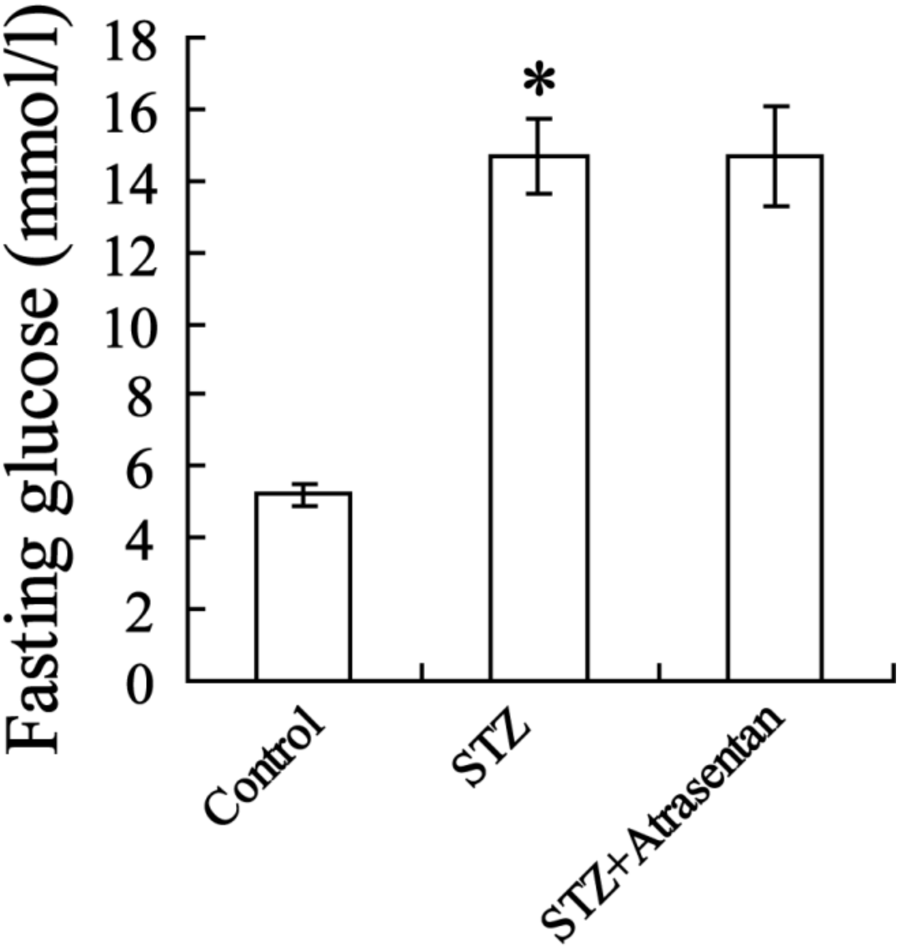

Suppl. 1 The effects of atrasentan on fasting blood-glucose of STZ-induced DN mice.
